# Ectopic Pregnancy with a Normally Located Levonorgestrel-Releasing Intrauterine System in a Woman with Adenomyosis: Case Report and Literature Review

**DOI:** 10.3390/jcm15010272

**Published:** 2025-12-29

**Authors:** Francesco Giuseppe Martire, Eugenia Costantini, Errico Zupi, Lucia Lazzeri

**Affiliations:** Department of Molecular and Developmental Medicine, Obstetrics and Gynecological Clinic, University of Siena, 53100 Siena, Italy; francescogmartire@libero.it (F.G.M.); zupi@unisi.it (E.Z.); lucialazzeri79@gmail.com (L.L.)

**Keywords:** ectopic pregnancy, adenomyosis, levonorgestrel-releasing intrauterine system, intrauterine system, case-report

## Abstract

**Background**: Ectopic pregnancy (EP) is a potentially life-threatening condition, often associated with acute abdominal pain and hemoperitoneum. Certain conditions, such as adenomyosis and the use of long-acting reversible contraceptives (LARC), may represent risk factors for the development of ectopic pregnancy. Management is tailored according to hemodynamic stability, reproductive desires, and associated comorbidities. **Case Presentation:** We report the case of a 39-year-old Caucasian woman with a history of adenomyosis and heavy menstrual bleeding (HMB) treated with a levonorgestrel-releasing intrauterine system (LNG-IUS). She presented to the emergency department with acute abdominal pain, vaginal bleeding, and a rising serum β-human Chorionic Gonadotrophin (β-hCG > 4000 mIU/mL). Transvaginal ultrasound revealed an adnexal mass (24 mm × 19 mm) consistent with a right tubal ectopic pregnancy, associated with hemoperitoneum. The patient, who expressed a desire for definitive sterilization, underwent laparoscopic bilateral salpingectomy. The procedure was uneventful with minimal intraoperative blood loss. Histopathological examination confirmed the diagnosis of right tubal ectopic pregnancy. **Literature Review:** A case report prompted a focused search of MEDLINE and Scopus (2015–2025) on ectopic pregnancy in users of levonorgestrel-releasing intrauterine systems. Eight eligible case-report studies assessing ectopic pregnancy type and device positioning were ultimately included. **Conclusions:** This case highlights the importance of early diagnosis of ectopic pregnancy, paying attention to any comorbidities, particularly adenomyosis, the role of minimally invasive surgery, and the possibility of adapting surgical management to the patient’s reproductive wishes.

## 1. Introduction

Adenomyosis is a benign uterine disorder characterized by the presence of endometrial glands and stroma within the myometrium, leading to uterine hypertrophy and hyperplasia. Once considered a condition of older women, it is increasingly recognized in younger, reproductive-aged patients due to advances in transvaginal ultrasound criteria and magnetic resonance imaging, which allow earlier and more precise diagnosis [1]. In young women, adenomyosis presents a clinical challenge: symptoms such as abnormal uterine bleeding and chronic pelvic pain can substantially impair quality of life [2,3,4]. At the same time, affected patients often require effective contraception without compromising future fertility, particularly in the context of rising maternal age; in Italy, for example, the mean age at first childbirth is now approximately 31.9 years, compared with a significantly lower age three decades ago [5]. Beyond its symptomatic burden, adenomyosis has relevant implications for reproductive health. Increasing evidence suggests that the disease is associated not only with impaired fertility and adverse obstetric outcomes, but also with alterations in uterine architecture and function that may influence early gestational processes [2,6]. These changes include disruption of the junctional zone, abnormal uterine peristalsis, and a modified inflammatory and hormonal microenvironment, which together may interfere with embryo transport and implantation dynamics. In this setting, the choice of contraception assumes particular relevance. Women with adenomyosis often require long-term, highly effective contraception that simultaneously provides symptom control while preserving reproductive options. The levonorgestrel-releasing intrauterine system (LNG-IUS) has therefore gained widespread acceptance as a first-line therapeutic strategy, owing to its dual role in reducing menstrual bleeding and dysmenorrhea while offering reliable contraception [7,8]. Nevertheless, the interaction between adenomyosis-related uterine changes and the local endocrine effects induced by LNG-IUS remains incompletely understood, particularly with regard to early pregnancy events.

Although ectopic pregnancy is a rare occurrence among LNG-IUS users, cases reported in the literature raise important questions regarding implantation mechanisms in altered uterine environments. Understanding these interactions is clinically relevant, as delayed diagnosis of ectopic pregnancy continues to represent a major source of morbidity. Against this background, the present case and accompanying literature review aim to contribute to a more nuanced understanding of ectopic pregnancy occurring in the presence of a normally positioned LNG-IUS in women with adenomyosis.

Long-acting reversible contraception (LARC), particularly the levonorgestrel-releasing intrauterine system (LNG-IUS), represents a therapeutic option [7]. The LNG-IUS alleviates bleeding and pelvic pain, may slow disease progression, and provides sustained contraception, with a favorable safety profile compared with systemic hormonal methods due to localized progestin delivery [8]. However, alternative medical and interventional treatments are increasingly available and should be considered according to symptom severity, reproductive goals, and patient preference.

Adenomyosis frequently coexists with endometriosis. Epidemiological studies report that, among women with endometriosis, the prevalence of adenomyosis ranges from 22% to 89% by transvaginal ultrasound and 27% to 65% by MRI [9,10]. Although endometriosis is known to impair fertility and tubal function, its direct association with ectopic pregnancy remains uncertain. Available evidence is limited and heterogeneous, and a causal relationship has not been conclusively demonstrated [11,12].

Ectopic pregnancy (EP) represents 1–2% of all pregnancies and remains a leading cause of first-trimester morbidity and mortality. Most ectopic implantations occur in the fallopian tube (around 90%), with the ampullary segment being the most frequent site; contemporary series report tubal locations in 85–95% of cases, reflecting case-mix and referral patterns. Recognized risk factors include prior tubal damage or surgery, pelvic inflammatory disease and assisted reproductive techniques [13,14]. Management is individualized according to a combination of clinical presentation, hemodynamic status, serum β-human Chorionic Gonadotrophin (β-hCG) and biochemical parameters, ultrasound findings, and should be personalized according to patient characteristics and disease severity [15].

In this context, we report a right tubal ectopic pregnancy in a woman with adenomyosis and prior LNG-IUS placement, successfully managed by laparoscopic salpingectomy.

## 2. Case Presentation

A 39-year-old Caucasian woman with a normal body mass index presented to the emergency department on 8 September 2025 with acute abdominal pain that resolved spontaneously, preceded by two days of vaginal bleeding and associated mastalgia and pelvic pain. Past medical history was notable only for diffuse adenomyosis and heavy menstrual bleeding, previously treated with combined oral contraceptives, a transdermal patch, and a vaginal ring; since February 2024 she had a levonorgestrel-releasing intrauterine system (LNG-IUS, 19.5 mg) in situ. Obstetric history included two spontaneous vaginal deliveries (2015, 2019). Family history was positive for ectopic pregnancy in the maternal grandmother.

**Figure 1:**
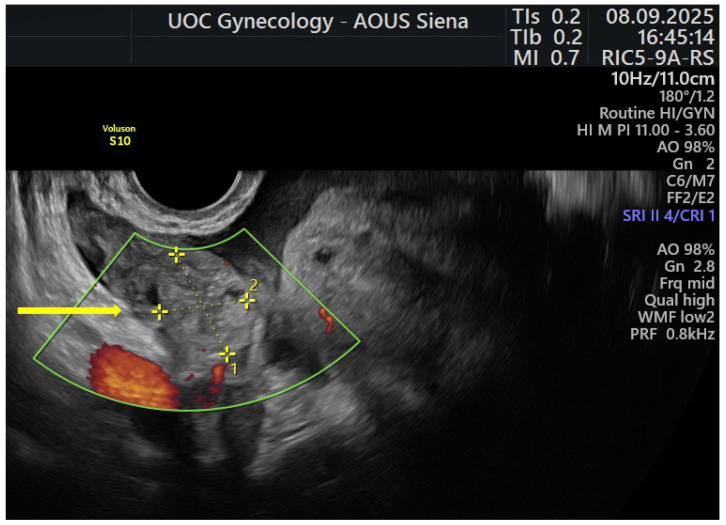
Transvaginal ultrasound showing a right adnexal hypoechoic rounded mass, separate from the ovary (arrow), consistent with the characteristic “blob sign” of early tubal ectopic pregnancy. The yellow dashed calipers (1 and 2) indicate the orthogonal ultrasound measurements used to assess the size of the adnexal mass.

On examination, the abdomen was soft with mild tenderness. An initial transabdominal ultrasound in the emergency department showed normal ovaries, a right corpus luteum, and a 28 mm hematic fluid collection in the rectouterine pouch. She was referred to gynecology, where transvaginal ultrasound demonstrated a normally sized, anteverted uterus with heterogeneous myometrium consistent with diffuse adenomyosis and the LNG-IUS correctly positioned. A right adnexal hypoechoic, rounded structure measuring 24 mm × 19 mm, separate from the ovary and consistent with the characteristic “blob sign”, was identified on transvaginal ultrasound (Figure 1, arrow), together with free hemoperitoneal fluid (anterior collection 36 × 27 mm; retrouterine collection 20 mm).

**Figure 2:**
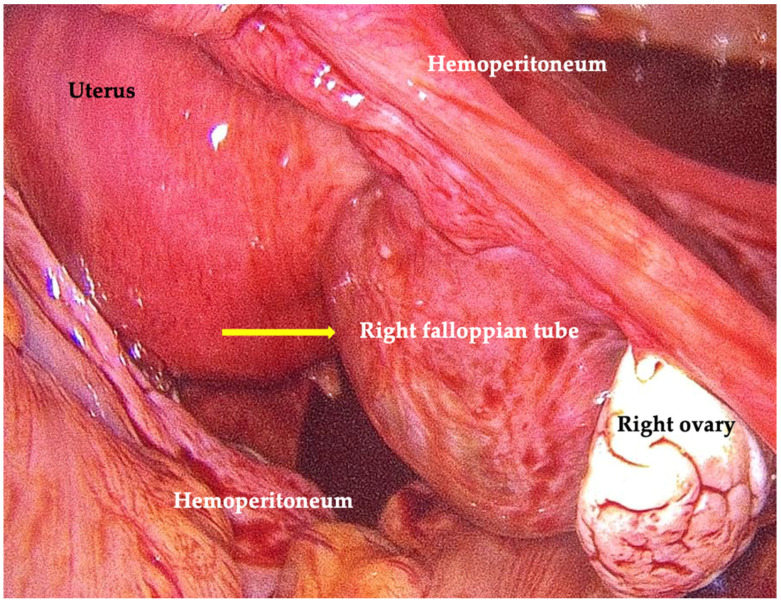
Laparoscopic view of the right fallopian tube enlarged and globular due to ectopic implantation (arrow), with associated hemoperitoneum.

She was admitted to the gynecology short-stay observation unit. Laboratory tests showed hemoglobin 12.7 g/dL and rising serum β-hCG concentrations (3953 → 4041 → 4337 mIU/mL within 24 h). Given the clinical and sonographic findings, minimally invasive surgery was recommended for suspected right tubal ectopic pregnancy. The patient also requested permanent contraception; therefore, bilateral salpingectomy was planned. Diagnostic–therapeutic laparoscopy revealed a globose uterus, normal left adnexa, a 3 cm bleeding ectopic pregnancy in the distal right fallopian tube, and approximately 300 mL of hemoperitoneum. A systematic inspection of the pelvic and abdominal cavity was performed, and no macroscopic endometriotic lesions or nodules were identified. A right salpingectomy was therefore performed to treat the ectopic pregnancy and a left salpingectomy for sterilization. The surgery was uncomplicated and blood loss was consistent with the procedure. Figure 2 shows a laparoscopic image of the right fallopian tube, enlarged and globular in shape, inside which the ectopic pregnancy was implanted. Histopathological examination confirmed ectopic gestation in the right fallopian tube. The postoperative course was uneventful.

The patient provided written informed consent for the processing of her personal data in accordance with current privacy regulations.

The case was managed in accordance with the 2013 Declaration of Helsinki.

## 3. Literature Review

**Figure 3:**
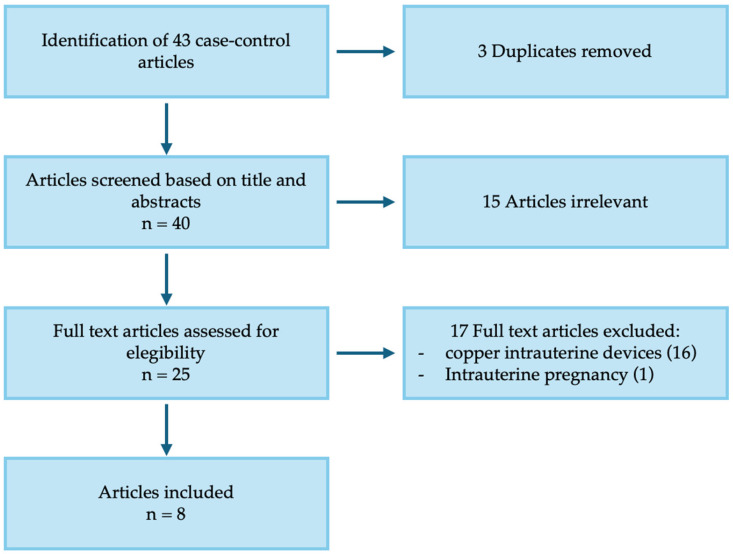
PRISMA diagram.

An electronic search of the MEDLINE database (via PubMed, U.S. National Library of Medicine) and Scopus was conducted to identify all English-language publications concerning ectopic pregnancy in patients using a levonorgestrel-releasing intrauterine system from January 2015 through October 2025 (Figure 3). The research question focused on identifying published case reports of ectopic pregnancy occurring in the presence of a levonorgestrel-releasing intrauterine system, in line with the clinical case observed at our center.

Relevant studies were retrieved using combinations of specific keywords and Medical Subject Headings (MeSH), including “ectopic pregnancy,” “intrauterine system,” “levonorgestrel-releasing intrauterine system,” “adenomyosis”.

Eligible sources consisted of case-report studies. Two independent investigators (F.G.M. and L.L) systematically reviewed all articles that met the predefined inclusion criteria.

A total of 43 studies were initially identified through PubMed and Scopus. After removal of three duplicates, 40 records underwent title and abstract screening, resulting in the exclusion of 15 irrelevant articles. Of the 25 studies considered potentially eligible, 16 were excluded because they focused on copper intrauterine devices rather than levonorgestrel systems and 1 was excluded because pregnancy was intrauterine. Ultimately, 8 studies were included in the final review (Figure 3).

Clinical characteristics of interest included the site of ectopic implantation, the levonorgestrel dosage of the intrauterine system, and whether the device was correctly positioned or displaced at the time of diagnosis. Additionally, the interval between LNG-IUS insertion and the diagnosis of ectopic pregnancy was evaluated, with a mean interval of approximately two years across the reviewed cases. In comparison, in the patient described in the present report, ectopic pregnancy occurred approximately 18 months after device insertion. These characteristics are summarized in Table 1.

The present case highlights several clinically relevant aspects. A first consideration concerns the rare but recognized risk of ectopic pregnancy among users of a levonorgestrel-releasing intrauterine system. The mechanism of action is worth recalling: its high contraceptive efficacy derives primarily from endometrial atrophy and a pronounced local inflammatory response, with cervical mucus modification contributing to a lesser extent. Unlike systemic hormonal contraceptives, however, ovulation is not consistently suppressed [24]. This explains why, in the uncommon event of contraceptive failure, the proportion of ectopic pregnancies is higher than in the general population. This phenomenon does not reflect an increased absolute risk of ectopic pregnancy due to the device itself, but rather the fact that LNG-IUSs reduce intrauterine pregnancies more effectively than ectopic ones; therefore, among the few “breakthrough” pregnancies that occur, the relative proportion of ectopic implantation is increased [25].

A thorough clinical history, careful physical examination, and transvaginal ultrasound performed by an experienced operator are essential to establishing the diagnosis and guiding management—whether medical or surgical. It is also important to assess the presence of concomitant conditions, such as endometriosis or adenomyosis, which may modify the risk of ectopic pregnancy and influence both diagnostic accuracy and therapeutic decision-making [26].

From a contraceptive standpoint, the 52 mg device demonstrates a first-year failure rate ≤ 0.2% (Pearl Index 0.1–0.2), with a cumulative 5-year pregnancy rate of approximately 0.7% and maintained efficacy for up to 8 years. The 19.5 mg and 13.5 mg systems show Pearl Index values of approximately 0.29 and 0.41, with cumulative pregnancy rates of 1–1.5% at 5 and 3 years, respectively [27]. Despite these reassuring data, sporadic cases of pregnancy—both intrauterine and ectopic—have been reported in the literature. Although the absolute risk of ectopic pregnancy remains very low, its proportion is higher among the small number of breakthrough conceptions. Moreover, lower-dose devices appear to be associated with a slightly higher risk, and the first two years after insertion represent the period of greatest vulnerability [18,28,29,30]. A large epidemiological study reported a higher incidence of ectopic pregnancy during the initial two years compared with longer durations of use (5.64/1000 vs. 2.25/1000) [31].

Unlike the copper intrauterine device—used primarily for contraception, with a first-year failure rate around 0.8% and a 10-year cumulative failure of approximately 1.9%—the LNG-IUS provides not only >99% contraceptive efficacy [32,33] but also substantial non-contraceptive clinical benefits. Continuous levonorgestrel release induces endometrial decidualization and marked atrophy, resulting in reduced heavy menstrual bleeding and dysmenorrhea, improvement in pelvic pain, and correction of anemia [34,35]. This same mechanism makes the LNG-IUS the first-line treatment for non-atypical endometrial hyperplasia, outperforming oral progestins in terms of histologic regression and bleeding profile [36].

Beyond clinical management considerations, ectopic pregnancy despite a correctly positioned LNG-IUS raises pathophysiological questions regarding implantation mechanisms beyond endometrial suppression alone. Subtle alterations in tubal transport or implantation dynamics may contribute to extrauterine implantation [24,25,30].

Although a direct causal link with ectopic pregnancy has not been demonstrated, the coexistence of adenomyosis-related endometrial alterations and local hormonal effects induced by LNG-IUS may define a modified implantation environment. This hypothesis remains speculative and warrants further investigation [2,6].

Adenomyosis and endometriosis warrant particular attention because of their implications for fertility and ectopic pregnancy risk. Adenomyosis may contribute to ectopic pregnancy by altering the structural and functional properties of the uterus. Infiltration of endometrial tissue into the myometrium can disrupt the junctional zone and normal uterine contractions, which may be associated with embryo transport through the fallopian tube. Local vascular and inflammatory changes may also reduce endometrial receptivity, favoring implantation outside the uterine cavity [2,37]. Although adenomyosis and endometriosis may coexist in a subset of patients, no macroscopic pelvic or peritoneal endometriosis was identified in the present case. The relationship between pelvic endometriosis and ectopic pregnancy has been discussed in the literature, mainly in the context of altered pelvic anatomy, inflammatory changes, or tubal dysfunction [6]; however, this association remains controversial. Available evidence is limited, largely retrospective, and heterogeneous, and does not allow definitive conclusions regarding a direct causal link between pelvic endometriosis and ectopic pregnancy. While chronic inflammation, pelvic adhesions, and tubal distortion have been proposed as potential mechanisms, these hypotheses have not been consistently supported by robust epidemiological or prospective data. Consequently, the contribution of pelvic endometriosis to ectopic pregnancy risk should be interpreted with caution and cannot be generalized, particularly in the absence of confirmed peritoneal disease. In this context, the present case highlights the occurrence of ectopic pregnancy in a patient with adenomyosis without associated pelvic endometriosis, underscoring the complexity of risk stratification and the need for further well-designed studies to clarify the clinical relevance of different endometriosis phenotypes.

Long-acting reversible contraceptives, particularly the LNG-IUS, are effective in managing symptoms and providing contraception. Although LNG-IUS does not appear to directly elevate ectopic pregnancy risk, its use in an altered uterine environment necessitates vigilant monitoring during early conception [38]. A history of previous gynecologic surgery (e.g., myomectomy, tubal surgery) may further contribute to this risk through adhesion-related distortion [39,40,41]. These considerations highlight the need for individualized counseling and follow-up to optimize reproductive outcomes and reduce the risk of ectopic pregnancy.

In the setting of adenomyosis, the 52 mg LNG-IUS is particularly effective: its antiprostaglandin and anti-inflammatory effects lead to a rapid reduction in menstrual flow (typically within 3–6 months), improvement of dysmenorrhea and chronic pelvic pain, frequent long-term amenorrhea, and, in many cases, the possibility of postponing or avoiding major surgical procedures [42]. Notably, among the case reports identified in our review, none described ectopic pregnancy occurring in patients with adenomyosis or reported this condition.

In recent years, additional therapeutic options for adenomyosis have emerged. Medical treatments such as oral GnRH antagonist combination therapies (e.g., relugolix–estradiol–norethisterone acetate) have shown efficacy in reducing pain and abnormal uterine bleeding, while uterine-sparing interventional approaches, including high-intensity focused ultrasound and microwave ablation, may represent alternatives for selected patients. These strategies are generally considered second-line options, reserved for women who are refractory to or unsuitable for first-line treatments. However, these strategies have specific indications and limitations, and their long-term outcomes—particularly in women requiring effective contraception or wishing to preserve fertility—are still under investigation [43,44,45].

The risk of pelvic inflammatory disease (PID) following IUS insertion must also be considered, although it is largely confined to the first 3 weeks post-insertion. PID may result in tubal damage and pelvic adhesions, both of which are established risk factors for ectopic pregnancy in subsequent gestations [46]. For this reason, a history of pelvic infections or suggestive symptoms should be carefully evaluated prior to insertion and reassessed in patients presenting with pelvic pain or a positive pregnancy test.

The diagnosis of ectopic pregnancy relies on the integration of transvaginal ultrasound findings and serial β-hCG measurements. In early pregnancy, pelvic pain and/or spotting should prompt evaluation for an adnexal extrauterine mass (occasionally featuring the characteristic “tubal ring/blob sign,” as in the present case), an extrauterine gestational sac, or free intraperitoneal fluid suggestive of hemoperitoneum. The absence of an intrauterine gestational sac in the presence of positive β-hCG is not, in itself, diagnostic. In uncertain cases, the condition is classified as a pregnancy of unknown location (PUL), requiring careful clinical, biochemical, and sonographic follow-up [13,47,48].

In our case, the presence of hemoperitoneum in conjunction with rising β-hCG levels necessitated surgical intervention. Minimally invasive laparoscopy allowed both diagnostic confirmation and definitive treatment, ensuring rapid postoperative recovery. The decision to perform bilateral salpingectomy addressed the acute condition while respecting the patient’s reproductive preferences, underscoring the importance of individualized care.

In clinical practice, the management of ectopic pregnancy is guided primarily by the overall clinical condition of the patient, ultrasound findings, and serum β-hCG levels. Although medical treatment with methotrexate may be considered in selected, clinically stable patients with low β-hCG levels and no evidence of tubal rupture, its use is relatively limited due to concerns regarding treatment failure and recurrence, particularly in the presence of underlying tubal pathology or previous pelvic inflammatory disease [49].

Surgical management therefore remains the most frequently adopted approach, and laparoscopy is preferred over laparotomy due to reduced blood loss, shorter hospitalization, decreased postoperative pain, and a lower risk of adhesion formation; laparotomy is reserved for situations of extreme instability, massive hemorrhage, or lack of resources/experience for minimally invasive surgery [48,50,51]. Salpingotomy is generally reserved for highly selected cases, as it carries a higher risk of persistent trophoblastic tissue and recurrence [52]. Importantly, the indication for surgical intervention is based on a combination of factors, including hemodynamic stability, visualization of an ectopic gestational sac with or without a yolk sac or embryo on ultrasound, the presence of hemoperitoneum, and β-hCG trends. Embryonic cardiac activity should be considered a marker of advanced ectopic gestation and reduced suitability for medical treatment, rather than a decisive criterion for surgery [26,53,54].

Multiple studies indicate that bilateral salpingectomy does not significantly impair ovarian reserve when utero-ovarian vascular pedicles are preserved [47,48]. Bilateral salpingectomy is considered safe with respect to ovarian function and confers potential benefits in cancer prevention [55]. A substantial proportion of high-grade serous carcinomas of the ovary and peritoneum originate in the fallopian tubes—particularly in the fimbrial region—via precursor lesions such as serous tubal intraepithelial carcinoma (STIC) [49,50,52]. This has led to increased consideration of opportunistic salpingectomy, especially in genetically high-risk women, as a risk-reducing strategy while preserving ovarian endocrine function until menopause [56,57,58]. It is important to note that other histotypes, such as endometrioid and clear cell carcinomas, are more strongly associated with endometriosis than with tubal origin [59,60]; consequently, the protective effect of salpingectomy alone for these subtypes is likely limited [61,62,63]. In summary, bilateral salpingectomy represents a safe procedure and a potential preventive strategy, to be incorporated into individualized counseling in accordance with current guidelines [64,65,66].

Ectopic pregnancy has been reported both in the presence of correctly positioned and displaced LNG-IUS devices. Current evidence does not allow definitive conclusions regarding the relationship between device position and the site of implantation.

Published evidence demonstrates that ectopic gestation may occur even when the device is documented as correctly positioned, including implantation within a previous cesarean section scar associated with a 52 mg LNG-IUS, confirmed by sonographic evaluation and surgical exploration [16]. Tubal ectopic pregnancies have also been reported in women with a fundally positioned LNG-IUS, reinforcing the need for prompt transvaginal ultrasound assessment following a positive pregnancy test because of the disproportionately elevated likelihood of extrauterine rather than intrauterine implantation [18,19]. Ovarian implantation has additionally been described in association with a 13.5 mg LNG-IUS, presenting with pelvic pain, rising serum β-hCG levels, and operative confirmation [22]. Rare extra-tubal presentations, including splenic ectopic pregnancy occurring with a low-lying LNG-IUS, have been characterized by hemodynamic instability and have required urgent surgical intervention [23]. In contrast, cases involving perforated, migrated, or non-visualized LNG-IUS devices show more advanced clinical severity, including ruptured tubal gestation, hemoperitoneum, salpingectomy, and retrieval of the displaced device [17,20,21]. Collectively, available reports indicate that any pregnancy occurring with an LNG-IUS in situ warrants immediate evaluation for possible ectopic implantation, while displaced devices exhibit a higher association with severe presentation and operative management [17,21,23]. Although LNG-IUS are widely used and risk factors for ectopic pregnancy are well recognized, few case reports describe ectopic gestation with a normally positioned device. Our case highlights this rare but clinically significant occurrence, emphasizing the need for careful evaluation and monitoring in women with adenomyosis using LNG-IUS. Further studies, including multicenter case series, are needed to clarify underlying mechanisms, identify patient-specific risk factors, and guide evidence-based counseling and management in this population.

## 4. Conclusions

Clinicians should recognize that when conception occurs in users of an LNG-IUS, the absolute risk of pregnancy remains very low, yet the relative proportion of ectopic implantation among contraceptive failures is increased. This underscores the importance of maintaining a high index of suspicion for ectopic pregnancy in women presenting with pelvic pain or abnormal bleeding while using an LNG-IUS, even when the device is documented as correctly positioned. In patients with adenomyosis, the diagnostic process may be further complicated by disease-related uterine changes and overlapping symptomatology. Although a direct causal relationship between adenomyosis and ectopic pregnancy has not been established, the present case illustrates how coexistence of uterine pathology and long-acting reversible contraception may influence clinical presentation and management decisions. Prompt evaluation using transvaginal ultrasound and serial β-hCG measurements remains essential, while treatment should be individualized according to hemodynamic stability, reproductive goals, and patient preference. Finally, this report highlights the need for further well-designed studies to clarify implantation mechanisms and identify patient-specific risk factors, thereby improving counseling, surveillance strategies, and outcomes for women with adenomyosis using LNG-IUS.

## Figures and Tables

**Table 1:** Summary of demographic and clinical characteristics of published case reports describing ectopic pregnancy occurring in the presence of LNG-IUS, including patient age, parity, relevant comorbidities, device dosage and position, site of ectopic implantation, and interval from device insertion. The present case is included for direct comparison.

References	Age (Years)	Parity	Clinical Data	LNG-IUS Dosage	IUS Position	Ectopic Pregnancy Location	Interval from LNG-IUS Insertion
Hitzerd, E., et al. (2018). [16]	36	Multiparous	Previous cesarean section	52 mg	In situ	Cesarean scar pregnancy	60 months
Howard, et al. (2017). [17]	31	Multiparous	Not specified	52 mg	Displaced	Left adnexa	54 months
Resta, C., et al. (2021). [18]	36	Multiparous	Not specified	52 mg	In situ	Left tubal	12 months
Singer, S. R., et al. (2023). [19]	31	Multiparous	Not specified	19.5 mg	In situ	Left tubal	36 months
Makena, D., et al. (2021). [20]	34	Primiparous	Not specified	52 mg	Displaced	Left tubal	24 months
Gaetani, S. L., et al. (2021). [21]	30	Multiparous	Not specified	Not specified	In situ	Right tubal	24 months
Panasowiec, L., et al. (2020). [22]	33	Multiparous	Not specified	13.5 mg	In situ	Right ovarian	Not specified
Antequera, A., et al. (2021). [23]	36	Multiparous	Not specified	Not specified	In situ	Spleen	4 months
Present case	39	Multiparous	Adenomyosis	19.5 mg	In situ	Right tubal	18 months

## Data Availability

The data that support the findings of this study are available from the corresponding author, E.C., upon reasonable request.
